# Academic Degree Bias Among Speaking and Leadership Roles at the American Academy of Orthopaedic Surgeons Annual Meetings, 2016-2021

**DOI:** 10.7759/cureus.56332

**Published:** 2024-03-17

**Authors:** Lucas Bartlett, Alton Daley, Adam Kazimierczak, Brandon Klein, Casey Humbyrd, Adam Bitterman, Randy Cohn

**Affiliations:** 1 Orthopedic Surgery, Northwell Health, Huntington, USA; 2 Orthopedic Surgery, Dartmouth-Hitchcock Medical Center, Lebanon, USA; 3 Orthopedic Surgery, Touro College of Osteopathic Medicine, Middletown, USA; 4 Orthopedics, University of Pennsylvania Perelman School of Medicine, Philadelphia, USA

**Keywords:** osteopathic, do, orthopedic surgery, bias, aaos, annual meeting

## Abstract

Objective: This study examined the proportion of Doctors of Osteopathic Medicine (DOs) across various speaking and leadership roles at recent American Academy of Orthopaedic Surgeons (AAOS) annual meetings.

Design: Meeting programs from the AAOS were publicly accessed and compiled between 2016 and 2021. Two categories of AAOS meeting participants were created. Invited speaker and faculty roles included moderators of symposia and program committee members while authors of presented papers were classified into academic roles. The proportion of DOs in each role type (invited speaker, academic) was recorded for each meeting program. The representation of DOs in these roles was then examined longitudinally across our analysis period using Pearson's Correlation.

Results: Overall, 1.1% (119/10,636) of all roles were held by DOs. Across our analysis period, DOs were disproportionately underrepresented as invited faculty or speakers (0.1%, 4/2791) compared with academic roles (0.1% vs 1.5%, p<0.001). Specifically, DOs were underrepresented as program committee members (0.08% vs 1.5%, p<0.001), symposia lecturers (0.1% vs 1.5%, p=0.004), and moderators of paper presentations (0.3% vs 1.5%, p=0.01).

Conclusion: Between 2016-2021, DOs were disproportionately represented as invited speakers or faculty at AAOS annual meetings. Our definition of diverse perspectives may need to expand to include osteopathic physicians.

## Introduction

Orthopedic surgery is one of the least diverse medical specialties [1-3]. As a result, the growing acknowledgment of gender and racial inequalities within the field has guided focused, organizational efforts to improve workplace diversity [4-7]. In 2018, the American Academy of Orthopaedic Surgeons (AAOS) operationalized a “renewed commitment to diversity” that subsequently led to notable increases in the number of women in AAOS leadership roles and society membership [7]. Although the growing recognition of gender and racial inequality has facilitated an increasing number of women and minorities in orthopedic surgery, other disparities are underrecognized [8].

As of 2021, Doctors of Osteopathic Medicine (DOs) comprised 5.9% of all practicing orthopedic surgeons, compared with up to 20% of primary care specialties [9]. In 2017, DOs comprised 5.5% of all practicing orthopedic surgeons [10]. In the preceding year, the Accreditation Council for Graduate Medical Education (ACGME) and the American Osteopathic Association (AOA) completed their consolidation of former allopathic and osteopathic residency programs under a single accreditation system [11]. However, the presence of a consolidated residency match was met with early speculation over its potential impact on osteopathic physicians [12-15]. Recent literature has validated some of these concerns with unique barriers for osteopathic medical students applying to orthopedic surgery and other surgical residencies which identify disproportionately lower match rates compared to allopathic medical students [15,16]. Although the literature has begun acknowledging the challenges faced by DOs entering orthopedic surgery, little evidence exists characterizing their representation as practicing surgeons or within academia.

Recent literature has examined the proportion of women speakers at orthopedic society meetings to highlight their underrepresentation [17,18]. Annual meetings are important platforms for knowledge sharing, establishing peer-to-peer connections, and promoting diversity [17,18]. Additionally, being able to serve in speaking or leadership roles offers physicians the opportunity to bolster their national reputation, develop leadership skills, and advance professional and/or academic rank [17,19,20]. As one of the largest gatherings for exchanging orthopedic knowledge, the AAOS annual meeting is a pivotal platform for orthopedic surgeons. To our understanding, no prior studies have attempted to quantify the representation of osteopathic physicians in faculty or speaking roles at annual AAOS meetings.

Our current study aims to examine the proportion of DOs in various roles at AAOS annual meetings between 2016 and 2021. Additionally, we seek to compare the proportion of DOs across a selection of "invited" versus "academic" roles to determine the equitability of role designation by academic degree. Although efforts have been made to improve diversity within orthopedics with respect to race and sex, osteopathic physicians remain historically underrepresented in the field. In its broadest definition, orthopedic societies advocating for workplace diversity should consider osteopathic physicians for speaking roles at their annual meetings.

## Materials and methods

Final programs from the AAOS annual meeting between the years 2016 and 2021 were publicly accessed by retrieving preliminary program PDFs, compiled, and independently reviewed by two authors (LB {DO}, BK {DO}). According to the previous methodology, the name and academic degree (MD and DO only) of faculty or speakers from a chosen number of program sections were gathered to partition surgeons across various "invited" and "academic" roles [17,18]. According to previously published methodology, faculty listed for instructional courses or resident-focused lectures, industry symposia, and business meetings (e.g., resolutions committees) were excluded [17,18]. Moderators of symposia, symposia lecturers, and program committee members were considered ‘invited’ speakers or faculty, while authors (first and last author) of presented papers were selected to represent those in "academic roles." Roles were partitioned into invited or academic categories based on their distinctive selection processes. While faculty appointment to "invited" roles is typically reserved for physicians with a proven academic track record, papers accepted for presentation are chosen from a generalized pool of submissions with selection being more dependent on study quality as opposed to academic prerequisites and reputation. Without bias limiting factors, we theorized that the proportion of DOs in academic roles should be proportional to those in invited roles.

For each year examined, physicians across all included roles were subcategorized by the subspecialty content area most related to their role. For program committee members and sections of paper presentations, subspecialty designation was previously listed in respective meeting programs. Symposia, however, lacked any formal assignments so subspecialty designation was independently categorized by two authors (LB {DO}, BK {DO}). If the subspecialty designation could not be ascertained from the listed symposia title, an internet query was performed with the name and title of the symposia moderator/lecturer to search for their respective subspecialty and was subsequently categorized. Among these sections, subspecialty assignment was determined from the titles of individual symposia and moderated sessions. Any discrepancies among reviewers required agreement from a third, independent reviewer (RC).

The proportion of DOs in each role was determined for each year. Proportions were represented collectively and by individual content areas for each year and role. These values were determined based on the number of DOs in each role type divided by the total number of positions available for each role type. This was repeated for all roles combined. To examine trends in representation over our analysis period, linear analyses using Pearson's correlation coefficient were performed for each individual role. Analyses were repeated for different combinations of role groupings, including the averaged group of invited roles and total roles. The proportion of DOs holding invited versus academic roles was then compared using a 2-tailed z-test for proportions. The proportion of DOs in academic roles was independently compared to each individual invited role and their collective average for each subspecialty. P-values less than 0.05 were considered statistically significant for all analyses.

## Results

A total of 10,636 physicians satisfied the inclusion criteria across our analysis period. Approximately 1.1% (119/10,636) of all roles were held by DOs (Table 1). For all collective roles, there were no significant trends in the representation of DOs for totaled or individual subspecialty content areas. When observing specific roles 0% (0/131) of symposium moderators, 0.08% (1/1199) of program committee members, 0.1% (1/963) of symposia lecturers, 0.3% (2/678) of paper presentation moderators, and 1.5% (116/7692) of paper authors were DO (Tables 2, 3). There were no significant trends in DO representation amongst program committee members (R=0.65, p=0.16), symposium lecturers (R=-0.39, p=0.44), symposium moderators (R=0, p=0.89), moderators of paper presentations (R=0.81, p=0.051), and authors of presented papers (R=0.81, p=0.051) for totaled subspecialties across our analysis period. Approximately 72% (83/116) of all DO authors recorded (first and last) were first authors. The proportion of DOs as first authors significantly increased over our study period (R=0.92, p 0.01).

**Table 1:** The number (%) of DOs in all roles for each subspecialty, between 2016 and 2021. R: correlation coefficient. No R values were statistically significant. DO: Doctor of Osteopathic Medicine.

Specialty	Total roles	Number (%) of DOs in all roles	R
Adult reconstruction	3089	34 (1.1%)	0.62
Foot and ankle	541	13 (2.4%)	0.60
Hand and wrist	477	11 (2.3%)	0.33
Pediatrics	619	13 (2.1%)	-0.01
Practice management	636	9 (1.4%)	0.70
Shoulder and elbow	1279	12 (0.9%)	0.15
Spine	977	4 (0.4%)	0.66
Sports medicine	1390	10 (0.7%)	0.02
Trauma	1276	12 (0.9%)	-0.70
Tumor and metabolic disease	352	1 (0.3%)	0.29
Total	10636	119 (1.1%)	0.79

**Table 2:** The number (%) of DOs serving in academic roles for each subspecialty, between 2016 and 2021. R: correlation coefficient. No R values were statistically significant. DO: Doctor of Osteopathic Medicine.

Specialty	Total academic roles	Number (%) of DOs	R
Adult reconstruction	2140	34 (1.6%)	0.63
Foot and ankle	432	13 (3.1%)	0.61
Hand and wrist	374	10 (2.7%)	-0.10
Pediatrics	449	13 (2.9%)	0
Practice management	452	9 (2%)	0.70
Shoulder and elbow	938	10 (1.1%)	0.15
Spine	722	4 (0.6%)	0.19
Sports medicine	980	9 (0.9%)	0.02
Trauma	953	12 (1.3%)	-0.70
Tumor and metabolic disease	242	1 (0.4%)	-0.13
Total	7682	115 (1.5%)	0.81

**Table 3:** The number (%) of DOs in all invited roles for each subspecialty, between 2016 and 2021. The correlation coefficient in bold is statistically significant. R: correlation coefficient, DO: Doctor of Osteopathic Medicine.

Specialty	Total invited roles	Number (%) of DOs	R
Adult reconstruction	949	0 (0%)	0.0
Foot and ankle	109	0 (0%)	0.0
Hand and wrist	103	1 (1.0%)	0.0
Pediatrics	170	0 (0%)	0.39
Practice management	184	0 (0%)	0.0
Shoulder and elbow	341	2 (0.6%)	0.0
Spine	255	0 (0%)	0.0
Sports medicine	410	1 (0.2%)	0.65
Trauma	323	0 (0%)	0.0
Tumor and metabolic disease	110	0 (0%)	0.0
Total	2971	4 (0.1%)	0.84

Overall, compared with academic roles (1.5%, 116/7692), DOs were disproportionately underrepresented as invited faculty or speakers (0.1%, 4/2791) across our analysis period (p<0.001) (Table 4). Additionally, DOs were disproportionately underrepresented as invited speakers or faculty across adult reconstruction (0% vs 1.6%, p=0.002), pediatrics (0% vs 2.9%, p=0.02), practice management (0% vs 2%, p=0.047), and trauma (0% vs 1.3%, p=0.04) content areas. When observing individual invited roles, DOs were disproportionately underrepresented as program committee members (0.08% vs 1.5%, p<0.001), symposia lecturers (0.1% vs 1.5%, p=0.004), and moderators of paper presentations (0.3% vs 1.5%, p=0.01). Across our analysis period, DOs were increasingly represented in all invited roles (R=0.84, p=0.03), however, no significant trends for specific categories of invited roles were observed.

**Table 4:** The proportion of DOs in academic versus individual and combined invited roles, between 2016-2021. Comparisons were performed utilizing a 2-tailed z-test for proportions with corresponding p-values shown. Comparisons were made independently for each individual invited role and their collective average. Significant p-values are marked in bold for reader feasibility and signify a higher proportion of DOs in academic roles versus the associated invited role and subspecialty. DO: Doctor of Osteopathic Medicine.

Specialty	Program committee members	Symposia lecturers	Symposia moderators	Moderators of paper presentations	Combined
Adult reconstruction	0.01	0.01	0.44	0.08	0.002
Foot and ankle	0.19	0.52	0.72	0.28	0.06
Hand and wrist	0.21	0.33	0.74	0.80	0.30
Pediatrics	0.19	0.16	0.58	0.28	0.02
Practice management	0.33	0.18	0.58	0.37	0.047
Shoulder and elbow	0.69	0.30	0.71	0.95	0.42
Spine	0.38	0.60	0.83	0.54	0.28
Sports medicine	0.20	0.87	0.66	0.37	0.17
Trauma	0.19	0.24	0.64	0.33	0.04
Tumor and metabolic disease	0.78	0.67	0.85	0.71	0.49
Total	<0.001	0.004	0.13	0.01	<0.001

## Discussion

While the emergence of national diversity directives has guided the growing recognition of gender and racial disparity, the underrepresentation of osteopathic orthopedic surgeons remains largely unacknowledged. Recent reports have highlighted the decreased opportunities for osteopaths seeking residency placement in the field, yet little evidence exists characterizing their representation as practicing surgeons or within academia [14,16]. The goal of the current study was to examine the proportion of DOs across a chosen selection of speaking or appointed faculty roles at AAOS annual meetings between 2016 and 2021. Additionally, to assess diversity among speakers or faculty as it pertains to academic degrees, we sought to compare the proportion of DOs across a selection of "invited" and "academic" roles.

Of the 10,363 roles examined, only 1.1% (119) were held by DOs. Based on recent census reporting from the American Association of Medical Colleges (AAMC), this percentage is considerably less than the 5.5% and 5.9% of DOs practicing orthopedic surgery in 2017 and 2021 respectively [9,10]. Despite an increasing practitioner population, the proportion of DOs in all observed roles did not increase significantly over our analysis period. Collectively, these findings highlight the underrepresentation of DOs as speakers overall at AAOS meetings. For physicians pursuing academic careers, the ability to serve in speaking roles at annual society meetings creates meaningful opportunities for academic, professional, and leadership advancement [5,17,18,21,22]. Generalized diversity within organizations has also been strongly correlated with greater performance, innovation, and financial success [20,21,23]. As such, constructing a diverse speaker population has critical implications for both surgeons and societies alike.

Additionally, we found DOs to be disproportionately underrepresented as invited faculty or speakers compared to those in academic roles (0.1% v 1.5%, p<0.001). Specifically, DOs were underrepresented as program committee members (0.08% vs 1.5%, p<0.001), symposia lecturers (0.1% vs 1.5%, p=0.004), and moderators of paper presentations (0.3% vs 1.5%, p=0.01). The relationship between inequitable speaking roles and gender imbalance in the senior faculty position has been supported by previous literature [5,24]. Similar to the ‘leaky pipeline’ phenomenon, the disproportionate representation of DOs as invited speakers or faculty may limit future opportunities to serve in high-ranking faculty or leadership positions [4,17,18,24]. Examinations of orthopedic society meetings have also suggested that limiting the roles available to underrepresented speaker populations creates unbalanced opportunities in gaining national visibility and constitutes a form of professional marginalization [17]. In our analysis, the gap in representation between submitted research and invited presentations indicates that external barriers may contribute to the lower-than-expected rates of DOs as invited speakers or faculty. Ultimately, the meritocracy of building a robust faculty pipeline should expand to meet the challenges faced by osteopathic physicians.

While the AAOS has been a leading advocate for improving workplace diversity, our results highlight the underrepresentation of osteopathic physicians at their annual meeting. Of the 1,199 program committee members observed over our six-year analysis period, DOs were represented just once. As diversity among meeting planning committees has been directly associated with diversity among conference speakers, the AAOS may consider appointing osteopathic physicians to such roles [18,23-25]. Entities such as the AAOS Diversity Advisory Board may also consider expanding their oversight or appointing subcommittees to create programs with equitable degree representation across session types. Transparent reporting of membership demographics is also critical for monitoring organizational diversity and understanding the longitudinal effects of such directives [17,18]. To our knowledge, the AAOS does not include the percentage of osteopathic members or their representation among organizational leadership positions in its annual report. In the future, the AAOS may consider internally monitoring the representation of osteopathic speakers at their annual meeting.

Despite the many similarities in medical training curricula compared with allopathic institutions, osteopathic medicine continues to be stigmatized by portions of the medical community [26,27]. Upon the consolidation to a single-match system, the accreditation standards set forth by the ACGME mandated significant program restructuring among former AOA orthopedic surgery programs [13,14]. Increased requirements in resident and faculty scholarly activity, expanded funding and shifts away from community center training sites comprise some of the changes required for accreditation under the ACGME [13,14]. Although the current unified match and single accreditation system are in their infancy, osteopathic medical students are still faced with inherent challenges to matching into orthopedic surgery. Barriers such as program eligibility, higher associated costs, and poor access to academic institutions may contribute to the disproportionately lower match rates observed for osteopathic medical students applying for residency in orthopedic surgery [14,15,16]. While the long-term effects have yet to be established, the integration of osteopathic physicians under the current single accreditation system creates theoretical opportunities for increasing academic productivity and cultivating professional relationships. Establishing these professional networks, which can influence annual meeting invitations, may help eliminate the discrepancy in osteopathic speaker representation at AAOS annual meetings. Given the association between academic productivity during residency and the pursuit of an academic career, increased research opportunities may also help osteopathic physicians build a robust faculty pipeline over time [28,29]. Ultimately, until the long-term effects of the single accreditation system can be determined, DOs may be unintentionally misrepresented as speakers at national society meetings or as academic faculty.

Limitations

This study has several limitations. Osteopathic physicians have the opportunity to present at the annual American Osteopathic Academy of Orthopedics which may be a select group that may be unaccounted for. The appointment to faculty positions or speaking roles requires acceptance from the individual selected. It is plausible those uninterested in academic medicine may be less inclined to accept the more exigent duties associated with invited-type roles. As such, the presence of selection bias may account for the lower-than-expected number of faculty or guest speaker positions held by DOs. Additionally, the partitioning of surgeons to a chosen number of invited and academic roles was performed according to previously described criteria and may be prone to bias. Lastly, program sections were also excluded according to the previous methodology, and we acknowledge our findings may not fully capture the representation of DOs at observed annual meetings.

## Conclusions

Compared to the percentage of Doctors of Osteopathic Medicine (DOs) practicing orthopedic surgery, osteopathic physicians were underrepresented in all observed roles at annual American Academy of Orthopaedic Surgeons (AAOS) meetings between 2016 and 2021. Additionally, DOs were disproportionately underrepresented as invited speakers or faculty, which highlights the need for improved role designation at annual AAOS meetings in order to more actively foster inclusivity and diversity in the field of orthopedic surgery. To ensure equal career opportunities, focused efforts should be made to monitor this issue during future society meetings and within the orthopedic community.
